# The Use of Coffee Residues as Sustainable Cultivation Substrates in Microbial Biotechnology: Up-to-Date Review and Future Perspectives

**DOI:** 10.3390/molecules31132382

**Published:** 2026-07-06

**Authors:** Aleksandra Piotrowicz, Agata Fabiszewska, Karina Jasińska, Katarzyna Wierzchowska

**Affiliations:** Department of Chemistry, Institute of Food Sciences, Warsaw University of Life Sciences-SGGW, Nowoursynowska 159c, 02−776 Warsaw, Poland; karina_jasinska@sggw.edu.pl (K.J.); katarzyna_wierzchowska@sggw.edu.pl (K.W.)

**Keywords:** coffee waste, spent coffee grounds, microbial bioprocessing, waste valorization, circular bioeconomy

## Abstract

The growing volume of agro-industrial and food-processing residues has intensified interest in their use as low-cost substrates for microbial bioprocessing. Coffee-derived waste streams, including spent coffee grounds (SCGs), wastewater, pulp, husk, and silverskin, represent abundant but still underutilized biomass resources. This narrative review evaluates their potential as liquid or solid substrates or as components of cultivation media for selected microbial systems, including microalgae, bioremediation- and bioprocess-related bacteria, edible fungi such as *Pleurotus* spp., and yeasts in the genera *Pichia*, *Kluyveromyces*, *Saccharomyces*, and *Yarrowia*. The review compares the suitability of individual coffee residues based on substrate composition, pretreatment requirements, inhibitory compounds, process limitations, and reported outputs. Coffee-derived residues can reduce substrate costs, support waste valorization, and partially replace conventional nutrients in microbial processes. However, their broader application is limited by compositional variability, conditioning or hydrolysis requirements, difficulties in process standardization, and downstream processing costs. Current evidence most strongly supports fungal cultivation on SCG-containing substrates, bacterial treatment of caffeine-rich wastewaters, yeast fermentation of hydrolyzed residues, and microalgal use of conditioned liquid streams. The review identifies key research gaps and outlines realistic directions for developing coffee-based microbial bioprocesses within a circular bioeconomy framework.

## 1. Introduction

### 1.1. Scale of Coffee-Waste Production

Coffee is one of the most widely consumed beverages worldwide, with annual production exceeding 165 million bags and global consumption continuing to increase [1]. The scale of this production generates substantial waste throughout the coffee value chain, including cultivation, primary processing, roasting, brewing, and retail. Consequently, the environmental and economic implications of coffee-derived residues have attracted growing scientific attention, leading to an increasing number of studies focusing on the quantification, characterization, and potential valorization of these waste streams [2]. During coffee production and processing, a significant portion of the original biomass is transformed into by-products and residual materials. It is estimated that approximately 40–45% of the mass of coffee fruits becomes waste during processing [3], resulting in the generation of millions of tons of biomass annually [3,4,5,6,7]. In recent years, global production of green coffee beans has reached approximately 10–10.5 million tonnes per year [4,7], with the largest contributions coming from Brazil, Vietnam, and Colombia, which together account for more than half of the global supply [4,7,8,9,10]. The scale of this production is directly linked to the generation of large volumes of waste across multiple stages of the coffee value chain. SCG is the dominant residue of coffee beverage preparation and instant coffee manufacture, whereas coffee pulp, husk, mucilage, and wastewater dominate earlier processing stages in producing regions. It is estimated that approximately 650 kg of spent coffee grounds are generated from each tonne of processed green coffee beans [3,6,11]. As a result, global production of spent coffee grounds is estimated at approximately 6–8 million tonnes annually [6,11,12].

In addition to spent coffee grounds, a variety of by-products are generated during the processing of coffee fruits. The predominant by-product is coffee pulp, the fleshy outer layer of the fruit, which accounts for approximately 37–45% of the total fruit mass.

Coffee husk comprises roughly 12–18% of the fruit mass, while parchment represents about 5.8–6.1%. Additionally, the roasting process yields coffee silverskin, which contributes approximately 4.2% [13] of the mass of roasted coffee beans [3]. The extensive production of coffee waste poses considerable environmental challenges. Upon disposal in landfills, spent coffee grounds (SCG) undergo biological degradation, releasing greenhouse gases, predominantly methane (CH_4_) and carbon dioxide (CO_2_). Estimates indicate that the carbon footprint associated with SCG disposed of in landfills reaches approximately 28.6 million tonnes of CO_2_ equivalent annually [6]. Furthermore, there are concerns regarding the toxicological impacts of coffee waste on ecosystems. This waste contains compounds such as caffeine, tannins, and polyphenols, which, at elevated concentrations, may have toxic effects on plant growth and microorganisms. Such substances have the potential to contaminate soil and groundwater through leaching processes [8]. The physical properties of this waste also pose significant environmental risks; dried SCG may pose a fire or ignition risk under inappropriate storage conditions, although this risk should be distinguished from the better-documented greenhouse gas emissions and leaching concerns associated with landfill disposal [4]. Additionally, various studies suggest that extracts from coffee waste may exhibit mutagenic and genotoxic effects in specific cellular models [14], potentially threatening aquatic organisms and underscoring the ecotoxicological significance of these extracts [6,10].

Conversely, the considerable biomass generated by the coffee industry is increasingly recognized as a valuable resource for energy recovery, biofuels, and bioactive compounds, aligning with the principles of a circular economy [3,6]. Current methods of coffee-waste management are evolving from traditional disposal practices toward more advanced circular-economy approaches. However, improper storage and handling of coffee residues still pose a significant environmental concern. Spent coffee grounds (SCG) are increasingly viewed as valuable secondary raw materials within the concept of a “coffee biorefinery,” rather than merely as waste products [11]. Consequently, various recovery and valorization pathways have been explored. One of the most common applications of SCG is in agriculture and horticulture, where it is used as compost, organic fertilizer, or soil amendment due to its nutrient content and organic matter [6,7]. Another important area of focus is energy production and biofuel generation. Given their relatively high calorific value, exceeding 20 MJ/kg, spent coffee grounds can be processed into fuel pellets and briquettes. Additionally, various chemical and biological conversion processes enable the production of biodiesel, bioethanol, biogas (methane), and bio-oil from this biomass [3,10,15].

Industrial and material applications also represent a growing area of interest. Coffee waste can be incorporated into the production of bioplastics and biocomposites, including reusable cups and biodegradable plant pots. Furthermore, SCG has shown potential as a low-cost sorbent capable of removing heavy metals and other pollutants from water environments [4,6,7]. Lastly, the food, cosmetic, and pharmaceutical industries are increasingly utilizing coffee residues as sources of bioactive compounds. Substances such as antioxidants, polyphenols, and caffeine can be extracted and used in dietary supplements, cosmetic formulations (e.g., creams and exfoliating products), and as functional food ingredients, particularly for their dietary fiber content [6,8]. Despite ongoing advances in waste valorization technologies, one of the primary barriers to the effective large-scale utilization of coffee waste remains the logistics of its collection and segregation. This challenge is particularly significant for dispersed sources such as households and small cafés, where systematic collection systems are often lacking, thereby limiting the feasibility of industrial-scale processing. Accordingly, this review aims to evaluate coffee-derived residues as potential substrates or components of media for microbial systems, considering their capacity to provide carbon, nitrogen, and macro- and micronutrients, as well as their technological and commercial feasibility.

### 1.2. Types of Waste Streams from the Coffee Supply Chain

The waste streams produced throughout the coffee supply chain demonstrate significant variability in both quantity and physicochemical composition, primarily influenced by the production stage and processing conditions. Generally, we can categorize these residues into two main types: primary waste, which occurs during the transformation of coffee cherries into green beans, and secondary waste, generated during the roasting process and beverage preparation [1,3]. This classification is essential, as it highlights not only differences in their origins but also consistent trends in chemical structure, functional properties, and potential valorization opportunities across the various waste fractions.

Primary waste (Table 1) is largely generated in coffee-growing regions and comprises several fractions that emerge during the process of separating the coffee bean from its fruit. Notably, coffee pulp constitutes the largest fraction, accounting for approximately 37–45% of the fruit’s total weight, especially in wet processing methods. In contrast, coffee husk, produced primarily during dry processing, makes up about 12–18% of the fruit’s mass, encompassing the outer layers of the dried fruit. Additional components, such as mucilage and parchment, develop during intermediate processing stages, with parchment forming a thin yet sturdy layer that envelops the bean. Although they are not always recognized as industrial by-products, coffee leaves and flowers offer a valuable source of biomass enriched with beneficial compounds such as caffeine and mangiferine [16,17]. A noteworthy characteristic of these primary residues is their high structural complexity and the predominance of lignocellulosic components, particularly cellulose and lignin, which contribute to the material’s rigidity, as evidenced in parchment [3,12].

Secondary residues (Table 1), generated mainly during roasting and consumption, include spent coffee grounds and silverskin, whereas coffee-processing wastewater should be treated as a primary processing liquid stream generated primarily in coffee-producing regions. Spent coffee grounds constitute the most abundant fraction, accounting for approximately 650 kg per tonne of processed green beans, reflecting the global scale of coffee consumption. Silverskin, a thin, papery layer covering coffee green beans released during roasting, accounts for approximately 4.2% of bean weight and is characterized by a high fiber and antioxidant content [13,18]. In addition, significant volumes of wastewater are generated, particularly during wet processing and demucilation stages. Some secondary residues, particularly SCG and silverskin, contain readily recoverable lipids, fiber, nitrogen-containing compounds, and bioactives. However, their direct use remains limited by moisture, inhibitory compounds, variability, and pretreatment requirements [3,6,11].

A critical parameter governing the suitability of coffee waste for biological processing is the carbon-to-nitrogen (C/N) ratio, for which a clear trend across waste categories can be identified. Spent coffee grounds exhibit a relatively stable C/N ratio ranging from 15:1 to approximately 22:1 [19,20], with nitrogen content between 1.18% and 4.0% and carbon content between 45% and 60% [4,9,19,20]. This places SCG close to the optimal range for microbial activity in composting and fermentation processes. In contrast, primary residues such as coffee pulp and husk typically achieve suitable C/N ratios only after composting, generally falling below 25:1 [21]. This indicates a broader trend in which secondary waste streams are more readily applicable to biological conversion processes without extensive pretreatment, whereas primary waste streams often require conditioning. In polymers such as cellulose and hemicellulose, carbon and nitrogen are structurally bound, making them less readily bioavailable than more labile organic compounds.

The distribution of bioactive compounds, particularly caffeine and polyphenols, also follows a distinct gradient across the waste streams. Coffee pulp is characterized by high concentrations, with caffeine content up to 31 mg/g and exceptionally high levels of total polyphenols (up to 442 mg GAE/g), as well as tannins (up to 17 mg/g). Coffee husk also exhibits significant tannin content, ranging from 18 to 93 mg/g [3,12]. In comparison, spent coffee grounds contain moderate amounts of caffeine, typically 1–2% (with reported values up to 7.6%), and approximately 1.0–1.5% total polyphenols, including chlorogenic acids [22]. Silverskin contains relatively low and variable caffeine levels, ranging from 0.70 to 53.30 mg/g [3]. This establishes a clear trend: primary processing residues, especially pulp, are richer in bioactive compounds, while secondary residues contain lower but still significant levels. Importantly, despite their beneficial antioxidant properties, these compounds may exert phytotoxic effects in their raw form, influencing their direct application in agriculture [11].

Most solid coffee-derived residues can be classified as lignocellulosic biomass, whereas coffee wastewater and digestates are liquid streams containing dissolved organic matter, nutrients, suspended solids, and inhibitory compounds. Coffee pulp is distinguished by its high lignocellulosic content, with cellulose ranging from 18 to 63%, hemicellulose from 2 to 29%, and lignin from 14 to 26% [23], making it particularly suitable for biochemical conversion processes such as bioethanol production [24]. In contrast, spent coffee grounds are dominated by hemicelluloses (15–42%) [25], rich in mannose and galactose, while containing lower cellulose (7–23%) and moderate lignin levels (0–26%) [26]. Coffee husk has a relatively high cellulose content (16–43%) but lower hemicellulose content, whereas parchment has the highest lignin content (up to 35%) and substantial cellulose levels (35–54%), reflecting its rigid, woody nature. Silverskin, although more heterogeneous, contains 10–24% cellulose and is notable for its exceptionally high dietary fiber content, reaching up to 72% [3,12,26,27]. These data highlight a structural trend in which higher lignin content corresponds to greater material recalcitrance and a greater suitability for thermochemical rather than biochemical conversion pathways.

Differences are also evident in lipid content: spent coffee grounds contain the highest levels, typically 10−25%, whereas primary residues such as pulp and husk contain only minimal amounts [3,9,24]. This supports a broader trend linking chemical composition with valorization potential. Specifically, SCG, due to their high lipid and hemicellulose content and favorable C/N ratio, are particularly suitable for biodiesel production and the recovery of mannose-rich sugars. In contrast, cellulose-rich pulp is better suited for fermentation processes for bioethanol production or for extracting antioxidant compounds. Materials with high lignin content, such as parchment, are better suited for thermochemical applications, including the production of solid biofuels such as pellets.

Overall, the data demonstrate that coffee-waste streams are highly heterogeneous but exhibit identifiable compositional and functional trends closely linked to their origin within the production chain, the coffee variety, and the processing method applied [7,15]. These trends not only determine their environmental impact but also define their optimal pathways for sustainable utilization, supporting their integration into circular economy models.

### 1.3. Why Are Coffee Residues an Interesting Substrate for Microorganisms?

Coffee, particularly in the form of spent coffee grounds (SCG), is a highly promising, low-cost, and readily available substrate for a wide range of microbial and biotechnological applications due to its complex, nutrient-rich chemical composition [11,12]. SCG contains a diverse array of macronutrients, including carbohydrates (up to 82%), proteins (10–18%), and lipids (2–24%), all of which play a crucial role in supporting microbial growth and metabolic activity [1,24]. The lignocellulosic structure of coffee residues may support enzyme-oriented bioprocesses. Still, this claim should be supported by studies of coffee-specific enzymes or clearly framed as an extrapolation from broader agro-industrial residues. Cellulose and hemicellulose can provide fermentable sugars after pretreatment, whereas lignin contributes to structural recalcitrance but may be transformed by selected ligninolytic fungi or valorized through non-fermentative routes [28,29]. Following appropriate processing, microorganisms can utilize sugars derived from this matrix, including glucose, galactose, and mannose, to synthesize a variety of high-value products, such as organic acids (e.g., lactic and succinic acids) and liquid biofuels (e.g., bioethanol) [7]. In addition, the relatively high lipid content (approximately 15%) makes SCG particularly suitable for the cultivation of oleaginous microorganisms, which can accumulate microbial lipids or produce biopolymers such as polyhydroxyalkanoates (PHA) [30].

Beyond its macronutrient composition, coffee waste also contains bioactive compounds, including caffeine, tannins, and polyphenols, which can exert inhibitory or toxic effects on many microorganisms, thereby limiting process efficiency. Nevertheless, certain specialized organisms, such as the fungi species *Pleurotus ostreatus* [31] or *Rhizopus oryzae* [32], possess the enzymatic capacity to biodegrade or biotransform these compounds into less toxic or even value-added metabolites, including antioxidants. The physicochemical properties of SCG, particularly its porous and fibrous structure, further contribute to its suitability as a substrate in solid-state fermentation (SSF) systems, where it serves as both a physical support matrix and a nutrient source for fungal growth, thereby enabling efficient substrate colonization and bioconversion [32,33]. Moreover, coffee residues have attracted attention for their potential prebiotic properties, as they may stimulate the growth of beneficial gut microbiota, such as *Lactobacillus*, and positively modulate microbial activity in the gastrointestinal tract. Additionally, the high organic matter content of SCG makes it an attractive substrate for anaerobic digestion, where complex microbial consortia decompose it to generate renewable energy carriers, including methane and biohydrogen [14,34].

Despite these advantages, the direct application of SCG is often constrained by its recalcitrant lignocellulosic structure and the presence of inhibitory compounds, necessitating the implementation of pretreatment strategies in many biotechnological processes. The rigid, complex architecture of cellulose, hemicellulose, and lignin limits direct microbial access, making it essential to employ pretreatment methods that disrupt this structure and enhance the availability of fermentable components. Such treatments typically involve partial hydrolysis of the biomass, thereby facilitating subsequent enzymatic degradation and the release of simple sugars, including glucose, galactose, and mannose, which are essential for microbial growth and the biosynthesis of target products such as bioethanol and PHA [3,5]. Concurrently, detoxification processes are often required to reduce the concentration of inhibitory compounds, particularly caffeine, tannins, and polyphenols, which can otherwise impair microbial metabolism and reduce overall process yields. These detoxification steps often involve preliminary extraction or transformation of these compounds, thereby improving fermentation performance and cultivation efficiency [11,26].

A wide range of pretreatment techniques has been investigated and applied at both laboratory and industrial scales, encompassing mechanical, chemical, physical, and biological approaches. Mechanical methods, such as grinding and shredding, are primarily used to increase the substrate’s surface area and improve enzyme accessibility [35]. Chemical treatments, including acid hydrolysis (e.g., using H_2_SO_4_) and alkaline hydrolysis (e.g., with NaOH), are among the most effective strategies for solubilizing lignocellulosic components and releasing fermentable sugars, as well as facilitating the recovery of lipids [12]. Physical methods, such as hydrothermal processing, steam explosion, ultrasonic treatment, and pressure-induced “popping,” further disrupt the biomass structure and enhance substrate reactivity [6]. In contrast, biological pretreatment methods, including composting [21] and solid-state fermentation with selected microorganisms, offer an environmentally friendly alternative by enabling the gradual degradation of complex polymers and the simultaneous detoxification of inhibitory compounds [3,15].

Certain exceptions exist depending on the specific application, particularly in the cultivation of edible mushrooms. *Pleurotus* spp. can tolerate and partially transform caffeine and phenolic compounds in coffee-derived substrates, but caffeine may inhibit mycelial growth, and high SCG proportions can delay or prevent fruiting. Therefore, SCG is generally more reliable as a substrate component than as an untreated sole substrate. However, even in these instances, applying a mild biological pretreatment, such as composting, has been shown to significantly enhance substrate quality and increase cultivation yields compared with using raw spent coffee grounds (SCG). Overall, while untreated spent coffee grounds can be used as a substrate in some processes, employing appropriate pretreatment strategies is generally essential to maximize nutrient bioavailability, reduce inhibitory effects, and ensure efficient and reproducible microbial or fungal bioconversion.

Therefore, this review aims to compare the suitability, limitations, technological maturity, and commercialization potential of coffee-derived residues as substrates or components of media for selected microbial systems.

## 2. Scope and Approach of the Narrative Review

This article is a narrative review focused on the use of coffee-derived residues as substrates or as components of media for microbial cultivation and bioprocessing.

The literature search was conducted in Scopus, Web of Science Core Collection, and PubMed/MEDLINE to identify peer-reviewed publications on coffee-waste streams, microbial systems, and biotechnological applications. The last literature search was performed on 11 April 2026. The bibliographic database search focused on the peer-reviewed literature. Patent documents, regulatory texts, and company websites were identified separately and used only as contextual sources.

Searches were performed using combinations of keywords related to coffee residues, microbial groups, and process applications. The main search terms included “spent coffee grounds”, “coffee wastewater”, “coffee pulp”, “coffee husk”, “coffee silverskin”, “coffee mucilage”, “microalgae”, “*Pseudomonas*”, “*Pleurotus*”, “yeast”, “*Saccharomyces*”, “*Pichia*”, “*Kluyveromyces*”, “Yarrowia”, “fermentation”, “bioremediation”, “cultivation medium”, “substrate”, “hydrolysate”, and “valorization”.

Example search strings included:

(“spent coffee grounds” AND “microbial substrate”);
(“coffee wastewater” AND (“microalgae” OR “algal cultivation”));
(“coffee wastewater” AND “*Pseudomonas*” AND “caffeine degradation”);
(“spent coffee grounds” AND (“*Pleurotus*” OR “oyster mushroom”));
((“coffee husk” OR “spent coffee grounds”) AND “*Saccharomyces cerevisiae*” AND “bioethanol”);
(“coffee waste” AND “yeast fermentation”);
(“spent coffee grounds” AND “biorefinery” AND “valorization”).

Original research articles were prioritized when they reported experimental data on substrate composition, pretreatment, microbial cultivation, process outputs, degradation efficiency, product formation, or technological limitations. Review articles were included if they provided broader context on coffee-waste composition, valorization pathways, circular bioeconomy models, food and feed applications, safety issues, or biorefinery concepts. Additional relevant publications were identified by screening the reference lists of key articles. However, existing reviews usually discuss coffee-waste valorization broadly, whereas fewer synthesize coffee-derived residues specifically as microbial substrates across microalgae, bacteria, fungi, and yeasts.

**Figure 1:**
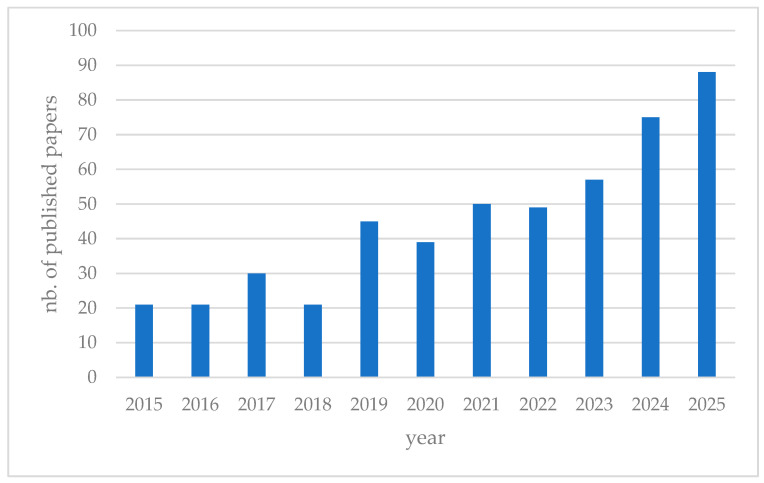
Annual number of publications related to coffee waste and coffee by-products according to Web of Science Core Collection records retrieved using the search terms “waste coffee” and “by-product coffee”. Search performed on 11 April 2026.

Studies were included when they addressed at least one of the following aspects: chemical or physicochemical characterization of coffee-derived residues; pretreatment or conditioning of coffee-waste streams; microbial cultivation or fermentation using coffee-derived substrates; biodegradation or detoxification of coffee wastewater; production of microbial biomass, enzymes, ethanol, metabolites, edible fungi, or other bioproducts; or technological, economic, regulatory, or safety aspects relevant to coffee-waste valorization. Studies were excluded if they focused exclusively on conventional coffee consumption, the sensory quality of coffee beverages, clinical or nutritional effects of coffee unrelated to waste valorization, non-biological material applications not relevant to microbial processing, or general biomass valorization without coffee-specific data. Non-English publications were not considered unless an English abstract or sufficient bibliographic information was available and the study was directly relevant to the topic (Figure 1). No strict lower publication-year limit was applied because older studies remain relevant for early applications of coffee residues in fungal cultivation and bioprocessing. However, priority was given to recent publications, particularly those from the last 10 years, during which the annual number of papers on coffee wastes and by-products increased by approximately 4.2-fold. The observed increase in publications indicates a growing research interest in coffee-derived residues as valuable bioresources rather than disposable wastes. This trend is likely associated with the increasing global generation of coffee by-products, rising environmental concerns related to landfill disposal and the leaching of inhibitory compounds, and the development of circular bioeconomy strategies. In this context, coffee residues are increasingly investigated as substrates for microbial cultivation, sources of bioactive compounds, and feedstocks for biorefinery-oriented processes.

Because this work was conducted as a narrative review rather than a systematic review, no formal PRISMA-based screening workflow, risk-of-bias assessment, or meta-analysis was performed. The available studies were heterogeneous in terms of substrate type, pretreatment method, microbial strain, cultivation conditions, process scale, and reported performance indicators. Therefore, quantitative pooling of results was not considered appropriate. Instead, the literature was analyzed qualitatively and organized thematically according to coffee-derived stream, microbial group, process objective, reported products, technological limitations, and maturity level.

During the preparation of this narrative review, AI-assisted tools were used solely to support the preliminary literature mapping, reference organization, summarization of selected publications, and language editing. Scopus AI (April 2026 operational state, Elsevier; accessed on 4 April 2026) and ResearchRabbit (web-based platform, accessed on 4 April 2026) were used to identify the relevant literature and citation relationships using representative topic-based queries, including “spent coffee grounds as microbial substrates”, “coffee wastewater bioremediation microorganisms”, and “coffee waste valorization bioprocessing”.

Google NotebookLM (accessed on 21 March 2026) was used for structured summarization of selected publications and extraction of key scientific information using representative prompts such as “summarize microbial applications of coffee residues”, “identify microorganisms used in coffee waste fermentation”, and “extract substrate composition, pretreatment methods, and bioprocess outputs”. Grammarly AI Writing Assistance (accessed on 24 April 2026) was used exclusively for grammar correction, language refinement, and improvement of readability.

All outputs generated using AI-assisted tools were critically reviewed, verified against the original literature, and edited by the authors. These tools were not used to generate scientific interpretations, conclusions, or research decisions. The authors take full responsibility for the final content of the manuscript.

Patent documents, company websites, and regulatory documents were analyzed separately from the peer-reviewed scientific literature. Patent records were used only to identify technological trends and examples of applied innovation, not as evidence of validated process performance. Company websites were used to provide examples of commercialization and business models, while regulatory documents were used to contextualize food- and feed-related safety considerations. These sources were therefore interpreted as supporting contextual material rather than as equivalent to peer-reviewed experimental evidence.

The narrative design of this review has several limitations. Because no formal systematic screening protocol was applied, the selection of studies may not capture all available publications on coffee-waste valorization. In addition, the reviewed studies differ substantially in substrate origin, pretreatment method, microbial strain, process scale, and performance indicators, which limits direct quantitative comparison. Patent records and commercial websites were used only as contextual sources and should not be interpreted as evidence of validated technological performance.

## 3. Coffee-Derived Residues as Microbial Substrates: Properties, Challenges, and Preparation

Coffee-derived residues can serve as microbial substrates because they contain organic carbon, nitrogen-containing compounds, lipids, minerals, and structurally bound polysaccharides. However, their suitability depends strongly on the type of residue and on the accessibility of these components to microorganisms. Solid residues such as spent coffee grounds (SCG), coffee pulp, husk, parchment, and silverskin are mainly lignocellulosic materials. In contrast, coffee wastewater and digestates are liquid streams containing dissolved organic matter and nutrients [2,3,25,36]. SCGs are particularly relevant as microbial substrates because they contain hemicellulose-derived sugars, proteins, lipids, and residual bioactive compounds. Their lipid fraction makes them attractive for biofuel-related processes, and their polysaccharide fraction can be hydrolyzed to fermentable sugars [3,12,25].

**Figure 2:**
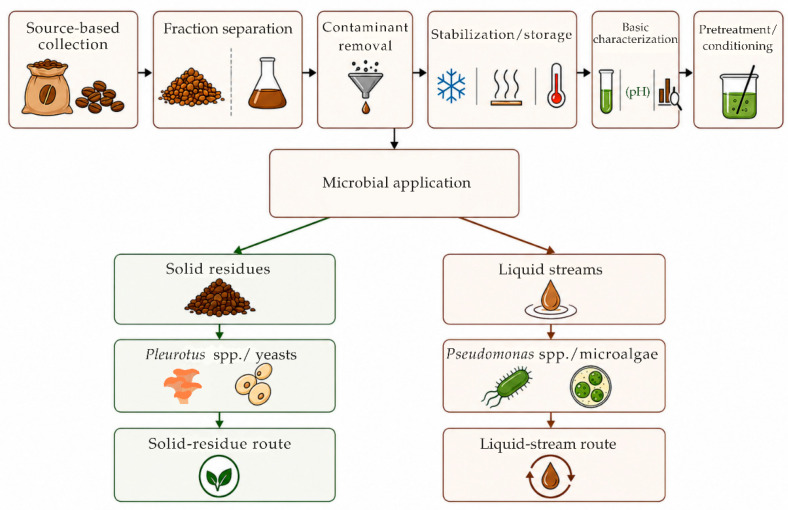
Simplified workflow for preparation of coffee-derived residues for microbial applications. Note: The scheme summarizes the main steps required before microbial use of coffee-derived residues. These include source-based collection, separation into major residue fractions, removal of non-coffee contaminants, stabilization and storage, basic physicochemical characterization, and application-specific pretreatment or conditioning. Solid residues are typically directed toward fungal and yeast-based processes after structural or biological conditioning. In contrast, liquid streams generally require clarification, dilution, pH adjustment, or detoxification before use in bacterial or microalgal systems. Created by the authors using Canva Web Application (Canva Pty Ltd., Sydney, Australia), live edition, accessed June 2026.

However, without pretreatment, a significant part of this carbon remains structurally unavailable because it is embedded in a lignocellulosic matrix [11,17,26,36]. Coffee pulp and husk are rich in lignocellulosic carbohydrates and phenolic compounds, making them suitable for fungal cultivation, enzymatic conversion, composting, and fermentation after appropriate conditioning. Coffee silverskin contains dietary fiber and antioxidant compounds, but its lower availability and variable composition may restrict large-scale use. In contrast, liquid streams such as coffee wastewater and digestates provide more directly available dissolved compounds and mineral nutrients. Still, their use is limited by acidity, high organic load, dark color, ammonium concentration, and residual inhibitory compounds [12,37,38,39]. Therefore, coffee residues should not be treated as a single uniform substrate category. Their microbial value depends on the balance between nutrient availability, inhibitory compounds, physical structure, moisture content, and the degree of pretreatment required before cultivation. Appropriate collection and separation strategies are essential because coffee-derived residues differ substantially in composition, moisture content, particle size, contamination risk, and potential end use [2,3,15]. Wet residues such as coffee pulp, mucilage, wastewater, and fresh SCG require rapid stabilization, refrigeration, drying, or immediate processing to prevent microbial spoilage [1,6]. Dry or fibrous residues such as husk, parchment, and silverskin should be stored under low-moisture conditions and separated from non-coffee contaminants [16,17]. Liquid streams require clarification, pH adjustment, and, where necessary, dilution or detoxification before microbial cultivation [37,38,40]. Solid residues intended for fungal or yeast-based processes may require grinding, sieving, hydrolysis, pasteurization, sterilization, or biological conditioning, depending on the target process [3,35]. A simplified workflow for preparing coffee-derived residues for microbial use is presented in Figure 2.

A generalized workflow for coffee-residue processing should include source-based collection from cafés, households, roasting facilities, or coffee-processing plants, followed by the separation of solid and liquid fractions, including SCG, pulp, husk, parchment, silverskin, wastewater, and digestate. Non-coffee contaminants should be removed before stabilization through drying, refrigeration, acidification, pasteurization, or rapid processing. Subsequent storage conditions should be adjusted to the moisture content and intended application of each residue. Before microbial use, basic characterization should include moisture, pH, C/N ratio, particle size, caffeine, phenolic compounds, and nutrient content [4,15,25], and for liquid streams, COD, BOD, suspended solids, and ammonium concentration [37,38,40]. Finally, application-specific pretreatment may involve grinding, sieving, hydrolysis, detoxification, pH correction, clarification, dilution, pasteurization, or sterilization. Such a standardized framework can improve reproducibility and help match each coffee residue with the most suitable microbial application.

Caffeine is a characteristic bioactive compound in coffee residues. Although some microorganisms can degrade or tolerate caffeine, many microbial systems require either dilution, adaptation, biological degradation, or partial removal of caffeine before efficient cultivation can occur. Fungal systems, including selected *Pleurotus* species [31,41], and caffeine-degrading bacteria such as *Pseudomonas* spp. [42]. They are especially relevant in this context because they can transform caffeine or tolerate coffee-derived inhibitory compounds more effectively than many non-adapted microorganisms [37,41].

Tannins, chlorogenic acids, and polyphenols are also important because they contribute to antimicrobial activity and substrate recalcitrance. At moderate concentrations, these compounds may serve as valuable antioxidant fractions, but in microbial cultivation, they can act as inhibitors unless they are extracted, diluted, degraded, or transformed during pretreatment. This dual role is important: the same compounds that make coffee residues attractive for antioxidant recovery [3,12] may reduce their direct suitability as fermentation substrates [25].

Melanoidins and other dark-colored compounds are particularly relevant in liquid coffee-derived streams. They contribute to color, residual chemical oxygen demand (COD), and limited biodegradability. In microalgal systems, dark color is especially problematic because it reduces light penetration and can suppress photosynthetic productivity. This makes decolorization, clarification, or dilution more important for algal cultivation than for many heterotrophic bacterial or fungal systems [38,40,43].

Low pH and high organic load are additional barriers in coffee wastewater. Acidic conditions may inhibit acid-sensitive microorganisms, while excessive COD or biochemical oxygen demand (BOD) can promote bacterial overgrowth, oxygen depletion, and unstable cultivation conditions. Similarly, digestates may contain useful nitrogen and phosphorus, but elevated ammonium concentrations, dark color, turbidity, and residual COD can restrict their direct use, especially in phototrophic cultivation [37,38,40,43].

In solid residues, the main structural limitation is lignocellulosic recalcitrance [2]. Cellulose and hemicellulose may serve as carbon sources, but lignin restricts microbial and enzymatic access to these polymers [35]. This is particularly relevant for husk, parchment, and other fibrous fractions [24], where pretreatment is often required to disrupt the matrix, release fermentable sugars, or improve fungal colonization [31,44,45].

Pretreatment is often required to convert coffee-derived residues from complex or inhibitory materials into usable microbial substrates. The objective of pretreatment depends on the target process: it may aim to increase surface area, release fermentable sugars, reduce inhibitory compounds, improve moisture and pH, remove suspended solids, decrease color, or enhance light penetration in phototrophic systems.

Mechanical pretreatment includes drying, grinding, milling, and sieving. These steps improve substrate homogeneity, reduce particle size, increase surface area, and facilitate enzymatic or microbial access to the material. Mechanical processing is particularly relevant for SCG, husk, pulp, silverskin, and other solid residues used in solid-state fermentation, enzymatic hydrolysis, or fungal cultivation [3,6,15,35].

Thermal and hydrothermal methods, including liquid hot water treatment and steam explosion, are used to disrupt lignocellulosic structure and improve the release of soluble sugars. Liquid hot-water pretreatment has been applied to SCG to improve bioethanol production without extensive detoxification, demonstrating that thermal processing can enhance substrate accessibility while reducing the need for harsh chemicals in some systems [46]. Steam explosion and related hydrothermal methods may also improve the digestibility of lignocellulosic fractions, although process severity must be controlled to avoid the formation of additional inhibitory by-products [6,15,26].

Chemical pretreatments include acid hydrolysis, alkaline treatment, and other solvent-based or catalytic approaches [12]. Acid hydrolysis is commonly used to release fermentable sugars from hemicellulose and cellulose, while alkaline treatment can help disrupt lignin-rich structures and improve enzymatic accessibility [12]. These methods can be effective, but they may require neutralization, washing, and detoxification before microbial inoculation, which increases process complexity [15,26,46,47].

Biological pretreatment includes composting, fungal pretreatment, and enzymatic hydrolysis [21,23]. These methods are generally milder and can reduce inhibitory compounds while improving substrate accessibility. Fungal pretreatment is particularly relevant for lignocellulosic residues because fungi can produce ligninolytic and cellulolytic enzymes. In mushroom cultivation, selected *Pleurotus* species can grow on coffee residues and partially degrade caffeine and phenolic compounds, although substrate formulation and conditioning remain important for stable yields [31,41,48].

Physical clarification and conditioning are especially important for liquid coffee-derived streams [37]. Filtration, centrifugation, settling, dilution, and clarification can reduce suspended solids, turbidity, and excessive organic load [49]. These steps are relevant for coffee wastewater and digestates, particularly when the target organisms are microalgae, because suspended solids and dark color reduce light availability [38,40].

Detoxification strategies aim to reduce the levels of caffeine, polyphenols, tannins, melanoidins, color, or other inhibitory fractions. Depending on the process, detoxification may involve biological degradation, adsorption, extraction, dilution, or electrochemical treatment. Electrochemical oxidation using a boron-doped diamond anode has been reported as a promising method to reduce color and COD in liquid digestate from coffee-waste biomass while retaining most of the ammonium nitrogen, which is beneficial for subsequent microbial cultivation [40]. Finally, pH adjustment is essential for coffee wastewater and digestates when their initial pH falls outside the tolerance range of the target microorganisms. For bacteria, yeasts, fungi, and microalgae, pH affects nutrient availability, enzyme activity, ammonium/ammonia equilibrium, contamination risk, and overall cultivation stability. Therefore, pH correction should be regarded as an essential step in substrate conditioning, since inappropriate acidity may limit nutrient accessibility, inhibit microbial growth, and reduce the efficiency of the intended bioprocess [37,38,40].

The suitability of coffee-derived residues for microbial cultivation depends on whether the target organism can access the available nutrients and tolerate or transform inhibitory compounds. Fungi are generally more suitable for solid lignocellulosic residues because of their enzymatic capacity to degrade complex polymers and phenolic compounds. Yeasts and bacteria usually require more readily available sugars or soluble substrates, so hydrolysis, extraction, or detoxification is often needed when using solid coffee residues. Microalgae are most relevant for liquid streams such as coffee wastewater and digestates, but their performance depends strongly on clarification, dilution, pH correction, and light availability [40,43].

**Figure 3:**
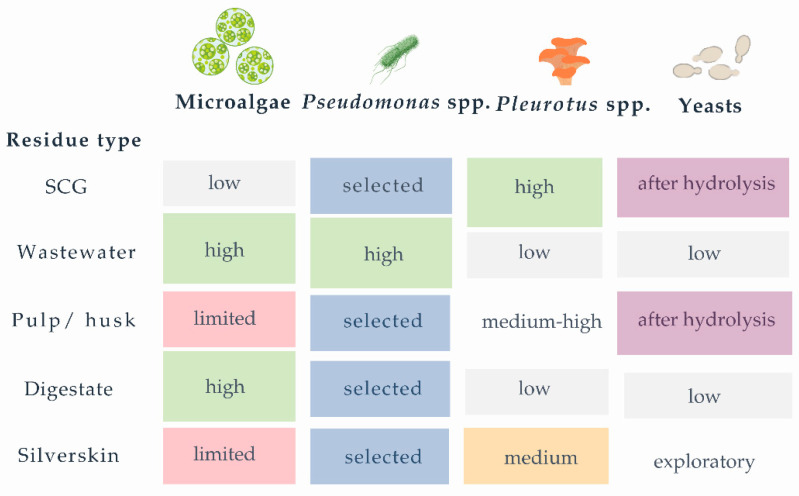
Comparative map of coffee-residue microbial valorization potential across selected microbial systems and residue types. Note: Cell labels indicate the relative suitability or maturity of each residue-microorganism combination, whereas colors are used only as visual aids for qualitative categories. “High” indicates strong applicability, “selected” indicates strain- or condition-specific relevance, “after hydrolysis” indicates dependence on prior sugar release, “limited” indicates restricted evidence or applicability, and “exploratory” indicates early-stage or concept-level validation. Created by the authors using Canva.

To complement the residue-specific comparison presented in Table 2, Figure 3 provides a simplified visual map of the relative suitability of major coffee-derived residues for selected microbial systems. The figure highlights that liquid streams are most relevant for microalgae and bacterial treatment, whereas solid and hydrolyzed residues are more suitable for fungal cultivation and yeast-based fermentation.

In summary, coffee-derived residues cannot be treated as interchangeable microbial substrates. Solid fractions generally require structural disruption or biological conditioning prior to fermentation, whereas liquid streams require clarification, pH adjustment, dilution, or detoxification before bacterial or microalgal cultivation. Thus, the choice of microbial system should be matched to the dominant limitation of each residue rather than only to its nutrient content.

## 4. Microalgal Cultivation on Coffee-Derived Liquid Streams

Among coffee-derived residues, liquid streams are the most directly relevant for microalgal cultivation because they can provide water, dissolved nutrients, and organic or inorganic nitrogen and phosphorus. Coffee processing wastewater and anaerobic digestates are therefore more suitable for algal systems than solid residues [38] such as husk, pulp, or spent coffee grounds (SCG), which usually require hydrolysis, extraction, or other preparatory steps before they can be used in liquid cultivation systems [2,3,12,15,36,40]. Coffee wastewater can serve as a liquid medium or remediation stream [37], whereas coffee-derived digestate can act as a nutrient-rich substrate, particularly due to its nitrogen and phosphorus content [39,40]. However, both streams are compositionally variable [37]. They may contain compounds or physical characteristics that restrict direct algal growth [39], including acidity, high organic load, dark coloration, suspended solids, residual chemical oxygen demand (COD) [40], ammonium accumulation, caffeine, and phenolic compounds [2,3,38]. In microalgal systems, conditioning is not only a general detoxification step but also a way to improve light availability [38,40,43]. Coffee-specific evidence is currently strongest for conditioned coffee-waste digestate. In contrast, broader conclusions regarding nutrient removal and pilot-scale algal wastewater treatment are supported primarily by general studies of digestate and wastewater [38,43,59].

The main functional value of these systems lies in the coupling of biomass generation with nutrient removal. During cultivation, microalgae can assimilate nitrogen and phosphorus into cellular biomass, while algal–bacterial interactions may contribute to partial reduction in organic matter. However, removal efficiency depends on strain selection, initial nutrient concentration, light regime, pH, hydraulic retention time, dilution rate, mixing, and reactor configuration. Therefore, nutrient removal values reported for wastewater-based microalgal systems should not be directly generalized to all coffee-derived streams without considering substrate composition and process conditions [38,43,59]. Coffee-based microalgal systems can be integrated into broader biorefinery concepts by combining effluent polishing with biomass production [60]. Depending on strain, cultivation conditions, and downstream processing, the generated biomass may be directed toward lipid-rich fractions for biodiesel [15], anaerobic digestion feedstock, fertilizer precursors, protein-rich biomass, pigments, or other co-products [3,43].

However, because the biomass is produced on waste-derived streams, not all downstream routes are equally realistic. Applications requiring high purity, strict safety control, or regulatory approval, such as food, feed, or nutraceutical-grade products [17,61]. It may be more difficult to justify using coffee wastewater or digestate as the cultivation medium [41,43]. In the near term, lower-risk routes such as bioenergy recovery, anaerobic digestion, soil amendment, or fertilizer-related applications appear more feasible than high-value food or nutraceutical uses [3,60]. Microalgal cultivation on coffee-derived liquid streams remains in the early stages of technological development. Most coffee-specific studies remain limited to laboratory or small-scale pilot systems [43]. Standardized operating conditions for coffee wastewater or coffee-derived digestates have not yet been established. Although wastewater-based microalgal cultivation is more advanced in the broader field, coffee streams introduce additional constraints, including variable composition [37], dark coloration [40], high organic load, acidity, suspended solids, ammonium accumulation, and inhibitory compounds [2,38].

From a practical perspective, industrial-scale application remains challenging because coffee-derived liquid streams are highly variable and often contain dark-colored compounds, suspended solids, high COD, residual caffeine, phenolics, and ammonium. These factors reduce light penetration, complicate culture stability, and increase the need for dilution, clarification, pH adjustment, filtration, decolorization, and contamination control. In addition, harvesting microalgal biomass on a large scale remains costly and energy-intensive. Therefore, although these systems are promising for coupling wastewater polishing with biomass generation, their coffee-specific application remains in an early technological stage and requires further pilot-scale validation.

The main advantage of these streams is their potential to partially replace synthetic nutrients while supporting wastewater polishing and biomass generation. However, this benefit is often reduced by the need for dilution, clarification, pH adjustment, filtration, decolorization, contamination control, mixing, aeration, and biomass harvesting. As a result, the key economic bottleneck is not nutrient availability itself but the cost of conditioning the medium and recovering biomass at acceptable productivity levels [38,40,43].

Overall, coffee-derived liquid streams offer a promising platform for coupling effluent treatment with microalgal biomass production [49]. However, their practical implementation remains constrained by variability in the medium [37], optical limitations, pretreatment costs, harvesting requirements [40], and the lack of coffee-specific pilot-scale validation [43]. Therefore, these systems should currently be described as promising early-stage biorefinery concepts rather than mature industrial technologies ready for broad deployment [2,3,60].

## 5. Bacteria in Caffeine-Rich Coffee Wastewater Bioremediation

In this chapter of the review, special emphasis is placed on *Pseudomonas* species. Among the microorganisms investigated for this purpose, *Pseudomonas* species have attracted particular attention due to their remarkable metabolic capabilities. One of the best-documented examples of a caffeine-rich bacterium is *Pseudomonas* spp. NCIM 5235, which has been evaluated for caffeine degradation in synthetic coffee wastewater. In synthetic coffee wastewater, *Pseudomonas* spp. NCIM 5235 completely degraded caffeine within 36 h using uninduced cells and within 2 h under optimized cell loading; however, these values should not be treated as directly transferable to real coffee-processing wastewater. Within this context, *Pseudomonas* spp. are particularly relevant due to their metabolic versatility and their established role in the degradation of methylxanthines and other organic pollutants. Their importance in coffee wastewater treatment, therefore, lies not only in general organic load reduction but especially in their ability to transform caffeine, one of the signature inhibitory compounds in coffee-derived liquid streams [37,42,51]. The same study also reported that caffeine degradation was not strongly affected by adverse pH and that induced cells could degrade theobromine present in wastewater, supporting the relevance of this strain for caffeine-rich coffee effluents [51].

The broader microbial literature indicates that caffeine degradation occurs primarily via N-demethylation and/or C-8 oxidation pathways. A recent review compiled known caffeine-degrading microorganisms and highlighted *Pseudomonas* as an important bacterial group involved in caffeine metabolism and bioremediation. These pathways are relevant because caffeine degradation does not merely remove the parent compound; it may also generate methylxanthine intermediates whose fate should be considered when evaluating treatment efficiency [62].

Other *Pseudomonas* strains also show caffeine-removal potential under controlled conditions. Still, degradation efficiency depends strongly on strain identity, induction state, caffeine concentration, pH, aeration [51], biomass loading, and the presence of additional organic compounds. Therefore, caffeine degradation by *Pseudomonas* should be described as feasible and mechanistically credible, but not automatically transferable across all coffee-wastewater matrices. From an engineering standpoint, the usefulness of *Pseudomonas* depends on whether degradation performance can be maintained beyond flask-scale screening. In the case of *Pseudomonas* spp. NCIM 5235, bioreactor studies showed that operational parameters such as aeration, agitation, biomass loading, and cultivation conditions strongly influence caffeine degradation and overall pollutant removal [42]. This shifts the focus from strain novelty to process control, emphasizing the need for operational parameters that support the scalability, reproducibility, and economic viability of bioremediation technologies. Optimization studies using experimental design methods further indicate that caffeine biodegradation in synthetic coffee wastewater is sensitive to cultivation parameters rather than being an invariant trait of the organism. Therefore, reported degradation times should not be interpreted as universal benchmarks. They are condition-specific results shaped by inoculum preparation, induction strategy, cell concentration, wastewater composition, oxygen transfer, and reactor operation [42,51].

For future process development, it will be important to move from synthetic model systems to real coffee-processing effluents and to adopt repeated-cycle or continuous operation. Real effluents may contain solids, polyphenols, pectins, sugars, fluctuating pH, and other compounds that can affect caffeine degradation, oxygen demand, biomass formation, and treatment stability. Pure-culture *Pseudomonas* systems are valuable for mechanistic studies of caffeine degradation and for identifying strains with strong methylxanthine-transforming capacity. Their advantage is interpretability: caffeine removal can be linked directly to the metabolism of a defined organism.

However, coffee wastewater is chemically complex, and caffeine removal alone does not ensure sufficient reductions in COD, BOD, suspended solids, nutrients, toxicity, or odor. The role of *Pseudomonas* in coffee-wastewater bioremediation should be described in two layers: first, as a mechanistically important genus for caffeine degradation; and second, as a possible member of mixed bacterial consortia designed for broader effluent polishing. For this reason, mixed bacterial consortia may be more suitable for real-world treatment of coffee wastewater. In mixed systems, different microorganisms can contribute complementary metabolic functions, including degradation of sugars, pectins, phenolics, nitrogen-containing compounds, and caffeine or caffeine-related metabolites. A mixed culture containing *Pseudomonas fluorescens* and *Escherichia coli* has been reported to reduce BOD, COD, and total solids in coffee-industry wastewater [63]. However, using *E. coli* would require careful biosafety assessment and is more relevant to contained wastewater-treatment systems than to product-oriented valorization [63]. It is also important to recognize that effective coffee-wastewater consortia do not necessarily have to include *Pseudomonas.* Pires et al. [37] selected an indigenous bacterial consortium composed of *Serratia marcescens*, *Corynebacterium flavescens*, and *Acetobacter indonesiensis*, which reduced BOD by 85%, COD by 60%, nitrogen and phosphorus by 80%, and ecotoxicity by more than 59% in coffee processing wastewater [37]. This supports the broader point that *Pseudomonas* spp. are particularly relevant for caffeine degradation, whereas whole-effluent remediation may benefit from broader consortia selected for multiple pollutant-removal functions.

## 6. Fungal Cultivation on Spent-Coffee-Ground-Containing Substrates

In cultivation practices, SCG plays diverse roles, ranging from serving as a primary substrate to being used as a nutritional supplement to optimize the carbon-to-nitrogen (C/N) ratio. An appropriate C/N ratio is generally considered important for balancing vegetative growth and fruiting, which, for this genus, should ideally fall within the range of 20:1 to 50:1 [45,64,65]. Furthermore, SCG can be used to produce innovative “coffee residue seeds” (CRS), which facilitate mycelial adaptation to the specific chemical composition of coffee, including caffeine, before the main growth phase [66]. Substrate preparation requires rigorous parameter control, including adjusting pH to 6.5–7.0 with gypsum or lime and pasteurizing (60–95 °C) or autoclaving (121 °C) to eliminate competing microorganisms, such as *Trichoderma* [41,44,45]. Comparative studies indicate that species and strain selection are critical. For example, Fan et al. reported better performance of selected *P. ostreatus* strains than *P. sajor-caju* strains on Brazilian coffee husk-based media. However, these results should not be generalized to all coffee-derived substrates [44].

The incorporation of coffee residues into the growth medium can significantly alter mycelial dynamics, leading to a dose-dependent retardation of colonization by metabolic inhibitors, primarily caffeine and phenolic compounds. Research indicates that the linear growth rate of species such as *P. floridanus* and *P. pulmonarius* decreases drastically as SCG concentration increases, falling from approximately 0.5 cm/day in control substrates to as low as 0.2 cm/day in media containing 40% SCG [48]. Complete inhibition of *P. ostreatus* mycelial growth has been observed at 2500 mg/L caffeine [44], while levels above 100 mg/L cause significant delays in colonization [44,48]. In some substrate formulations, particularly those with insufficient structural material, SCG proportions above approximately 30% may retard colonization or inhibit fruiting due to high levels of phenolic and caffeine, as well as substrate compaction [48]. However, higher proportions of coffee grounds may still support productive cultivation when combined with suitable structural materials, such as grasses, as shown by high biological efficiency in optimized coffee-ground/grass mixtures [65]. Although reducing particle size can increase surface area and improve microbial access, excessively fine SCG particles may promote substrate compaction and restrict oxygen circulation. Therefore, a universal optimal particle-size range for SCG alone has not yet been clearly established in coffee-based *Pleurotus* cultivation systems. Available studies indicate that high biological efficiency is more reliably achieved by combining fine SCG with structural lignocellulosic materials, such as wheat straw or grasses cut into approximately 2–5 cm segments, which improve porosity, aeration, and gas exchange during fungal respiration. Such structural adjustment can reduce compaction and support higher biological efficiency in optimized SCG-containing substrates [45,65]. Among the species tested, *Pleurotus djamor* has been identified as the most precocious, capable of forming primordia in as little as 11–13 days [67]. Species and strain selection also play an important role. Comparative studies have shown that different *Pleurotus* species respond differently to coffee-derived substrates. For example, Fan et al. [44] observed better performance of a selected *Pleurotus ostreatus* strain than other tested *Pleurotus* strains on Brazilian coffee husk, while Salmones et al. [67] reported differences in biomass production and substrate degradation among *Pleurotus* species cultivated on coffee pulp and wheat straw. These findings indicate that the suitability of coffee-based substrates should be evaluated for each species, strain, and residue type rather than generalized across the whole genus.

The genus *Pleurotus* possesses unique biological detoxification capabilities, utilizing powerful extracellular enzymes, such as laccase and the manganese-dependent peroxidase (MnP), to transform coffee waste. The degradation of caffeine proceeds through a sequential N-demethylation pathway, yielding metabolites such as theophylline (1,3-dimethylxanthine), paraxanthine, and theobromine, which are eventually converted into xanthine [41,48]. This process begins during the vegetative growth phase and results in a reduction in caffeine content in the substrate by 60% to over 91%, while toxic phenolic compounds are reduced by 21% to 79% [44,48]. These enzymatic processes contribute to the transformation of lignocellulosic substrates and may reduce the concentration or bioavailability of inhibitory compounds. However, contamination control still depends primarily on proper substrate preparation, hygienic handling, and appropriate pasteurization or sterilization procedures [41,45]. Some studies report degradation and conversion of caffeine, whereas others indicate that part of the caffeine may be absorbed or accumulated in fungal tissues rather than fully degraded [41,44,66]. This suggests that the fate of caffeine depends on the fungal strain, the type of coffee residue, the substrate composition, and the cultivation conditions. The high degree of bioconversion achieved during cultivation allows the process to be characterized as mycoremediation or waste valorization, transforming problematic urban and industrial residues into potentially valuable biomass and spent substrate with possible secondary applications [41].

The biological efficiency (BE) of *Pleurotus* spp. on coffee-enriched substrates is highly competitive, reaching peaks of 106.6% [65] in optimal mixtures of 50% SCG with grasses and approximately 105% in combinations with wheat straw [64]. Cultivation on coffee-based substrates may modify the chemical composition of fruiting bodies, including carbohydrate, lipid, mineral, and fatty-acid profiles. However, potential sensory effects should be described cautiously unless supported by dedicated sensory analysis [48,64,65]. These compositional characteristics may support their nutritional value and potential use in low-fat, energy-conscious diets. They offer a favorable fatty acid profile dominated by polyunsaturated linoleic acid (up to 83%) [48] and are rich in minerals such as magnesium, iron, and potassium [48,64].

Low caffeine levels in *P. ostreatus* fruiting bodies have been reported in SCG-based cultivation systems, and Carrasco-Cabrera et al. estimated that very large amounts of fresh mushrooms would be required to reach the caffeine content of a single espresso [41]. However, caffeine accumulation may vary with cultivation method and coffee residue type, with higher levels reported in mushrooms grown from coffee residue seeds [66]. In coffee husk-based cultivation, Fan et al. reported reduced caffeine and phenolic contents and increased protein concentration after *Pleurotus* cultivation, suggesting the potential use of the residual substrate as ruminant feed after appropriate safety evaluation [44]. More broadly, spent mushroom substrate from coffee-based systems may also be considered for composting, organic fertilizer production, or bioenergy applications [45].

The use of SCG in mushroom cultivation is particularly attractive at small and local scales, where coffee waste can be collected from households, cafés, or food-service systems and converted into edible biomass or other value-added products [41,45]. However, several barriers remain to large-scale industrial adoption, including the high energy costs associated with substrate sterilization and the need to use elevated spawning rates of up to 20% by weight to overcome initial growth inhibition on tougher residues like coffee husks [44]. Despite these challenges, the model aligns with circular economy strategies by minimizing the carbon footprint of food production through localized waste valorization [41,65]. A significant post-harvest challenge is the high perishability of Pleurotus ostreatus mushrooms due to their lack of a protective cuticle and their high respiration rates (200–500 mg CO_2_/kg/h); they maintain quality for 8–11 days at 0 °C but only 1–2 days at 20 °C. To extend shelf-life, strategies such as modified atmosphere packaging (MAP) and the application of edible coatings, such as chitosan or alginate enriched with essential oils from lavender or peppermint, are recommended to inhibit enzymatic browning and pathogen development [45].

Overall, SCG should be considered a valuable yet technically demanding substrate component for *Pleurotus* cultivation. Their successful use depends on appropriate dilution with structural materials, control of moisture and aeration, reduction in the risk of contamination, and careful selection of fungal species and strains. When these factors are optimized, coffee-based substrates can contribute not only to mushroom production but also to enzymatic valorization, spent-substrate reuse, and broader circular-economy applications [41,45,48,64,65].

## 7. Yeast-Based Fermentation of Hydrolyzed Coffee Residues

Yeasts are integral members of coffee-associated microbial communities, particularly during wet fermentation and in sugar-rich coffee side streams. Their significance extends beyond their contribution to coffee quality because selected yeasts can also ferment hydrolysates or extracts derived from coffee residues into ethanol, aroma-rich beverages, and value-added metabolites. The review literature supports the dual role of yeasts as both natural participants in coffee processing and potential biotechnological agents for residue valorization [68]. In the context of coffee-waste valorization, yeasts are particularly relevant for residues that contain soluble sugars or can be converted into fermentable sugars after pretreatment. Coffee mucilage and some liquid fractions may be more directly fermentable. In contrast, lignocellulosic residues such as spent coffee grounds (SCG), coffee husks, and pulp usually require hydrolysis or other conditioning steps to enable efficient yeast fermentation. Coffee-derived residues differ substantially in their suitability for yeast fermentation. Sugar-rich streams, such as mucilage or selected aqueous extracts, can be used more directly, whereas lignocellulosic fractions require pretreatment to release fermentable sugars. SCG and coffee husk are particularly relevant because they contain carbohydrate fractions that can be converted into glucose, mannose, galactose, and other sugars. Still, their direct use is limited by their recalcitrant structure and the presence of inhibitory compounds [69]. Recent work on SCG has demonstrated that liquid hot water pretreatment, followed by separate hydrolysis and fermentation, can support ethanol production without an additional detoxification step, reinforcing the practical relevance of SCG as a second-generation bioethanol feedstock [46]. Coffee husk hydrolysates have also been successfully fermented by *Saccharomyces cerevisiae*, confirming that residues from earlier stages of the coffee chain can serve as fermentation substrates after suitable hydrolysis [70].

To clarify the current evidence base, selected yeasts relevant to coffee-residue fermentation are summarized according to substrate type, required conditioning, main products, and coffee-specific maturity (Table 3).

The comparison highlights that most yeast-based routes depend on hydrolyzed or otherwise conditioned coffee residues; therefore, reported fermentation performance should be interpreted in relation to pretreatment efficiency, inhibitor management, nutrient supplementation, and downstream recovery requirements. In this context, *Saccharomyces cerevisiae* and *Pichia kluyveri* currently have stronger coffee-specific experimental support [71], whereas *Kluyveromyces marxianus* [68,72] and *Yarrowia lipolytica* [12,73] are better framed as promising yeasts whose broader biotechnological traits still require validation in coffee-derived matrices.

In addition to monoculture yeast fermentation, mixed systems are emerging as an interesting option for modifying SCG hydrolysates. For example, SCG hydrolysates have been transformed using *Lachancea thermotolerans* and the lactic acid bacterium Lactiplantibacillus plantarum to develop novel alcoholic beverages, demonstrating that yeast-based valorization can also overlap with mixed-culture, beverage-oriented fermentation [56]. This direction is useful when sensory profile, acidity, and aroma modulation are as important as ethanol yield. The cited study specifically evaluated the modification of SCG hydrolysate by a non-*Saccharomyces* yeast and a lactic acid bacterium.

*S. cerevisiae* remains the most investigated yeast platform for ethanol production from coffee-derived residues. Its main advantages are its established industrial use, high ethanol tolerance, and strong performance in fermentable sugar-rich hydrolysates. In coffee-waste systems, however, its efficiency depends strongly on pretreatment, sugar release, inhibitor management, and nutrient supplementation. The most established product from the *S. cerevisiae*-based conversion of coffee residue is second-generation bioethanol. Divyashri et al. reported apparent ethanol yields of 0.59 and 0.83 g/g of reducing sugars for free and encapsulated cells, respectively; however, because these values exceed the classical stoichiometric yield from hexoses, they should be interpreted with caution and not directly compared with conventional ethanol yield coefficients without clarification of the calculation basis. These data support the feasibility of coffee husk as a fermentation substrate after hydrolysis and show that an immobilization strategy can materially influence process performance [70].

SCG-derived substrates can also support higher-value yeast processes. Spent coffee grounds extract has been used for the co-production of glutathione and 5-aminolevulinic acid by *S. cerevisiae* NMZ−2, indicating that coffee residues may serve not only as bioethanol feedstocks but also as substrates for specialty metabolites [74]. This is important because specialty bioproducts may offer stronger economic potential than bulk fuels.

*Non-Saccharomyces* yeasts contribute to the diversification of coffee-waste bioprocesses. *Pichia kluyveri* has been used to ferment SCG hydrolysates into low-alcohol beverages while modifying the volatile and non-volatile composition. Yeast-extract supplementation increased ethanol production from 1.47% to 1.98% (*v*/*v*). It improved the formation of aroma-active esters, making *P. kluyveri* particularly relevant when product quality and aroma modulation are more important than maximal ethanol titer alone [71].

*Kluyveromyces marxianus* should be treated with greater caution. This species is broadly recognized as fast-growing, thermotolerant, and attractive for food and lignocellulosic bioprocessing. Still, the evidence base for coffee-derived residues remains less developed than that for *S. cerevisiae* or *P. kluyveri*. Therefore, *K. marxianus* is better described as a promising future platform for converting coffee residue rather than as a dominant yeast in the current coffee-waste literature [68,72]. *Yarrowia lipolytica* is an oleaginous, strictly aerobic, non-conventional yeast known for lipid metabolism, growth on hydrophobic substrates, and production of enzymes, organic acids, microbial lipids, and other metabolites. These properties make it conceptually relevant for lipid-rich or hydrophobic waste streams, including SCG-derived fractions. Still, direct evidence for *Y. lipolytica* in coffee-waste fermentation remains limited compared with *S. cerevisiae* and *P. kluyveri*. Existing coffee-related work shows that *Y. lipolytica* can modify volatile and non-volatile profiles during coffee fermentation, especially in green coffee, but this is not equivalent to validated SCG-waste valorization [75]. For this reason, *Y. lipolytica* should be considered an emerging candidate for future bioprocessing of coffee residue, particularly when targeting lipid-rich fractions, hydrophobic substrates, or aroma-active transformations. Its broader relevance is supported by reviews that describe *Y. lipolytica* as a versatile biorefinery platform capable of utilizing complex, low-cost substrates, effluents, and solid waste, but coffee-specific validation is still needed [73].

Other oleaginous yeasts may currently have stronger evidence specific to coffee waste than *Yarrowia*. For example, a recent SCG biorefinery study used SCG hydrolysate in fed-batch fermentation with *Rhodosporidium toruloides*, producing microbial oil and carotenoids after prior extraction and pretreatment steps. This supports the broader relevance of oleaginous yeasts for coffee-residue valorization, even if the organism is outside the main *Saccharomyces*/*Pichia*/*Kluyveromyces* framing [76].

Yeast-based coffee-residue valorization can generate several product categories, including ethanol, low-alcohol beverages, aroma-related compounds, microbial biomass, glutathione, 5-aminolevulinic acid, microbial oil, and selected specialty metabolites. However, the maturity of these routes differs substantially. Bioethanol production by *S. cerevisiae* from hydrolyzed residues is the most established route, while beverage-oriented fermentation with *non-Saccharomyces* yeasts and specialty metabolite production remain more exploratory.

Fermentation efficiency depends strongly on substrate preparation. Sugar release, inhibitor management, nutrient balance, sterility, oxygen availability, and downstream recovery all influence technical feasibility and cost. In SCG valorization, especially pretreatment and hydrolysis, often account for the majority of the process burden. Therefore, promising yields reported in laboratory studies should not be interpreted as direct evidence of commercial readiness [3,10,46].

The main advantage of yeast-based systems is product flexibility. The same coffee-derived residue stream may be directed toward ethanol, beverages, biomass, or specialty metabolites depending on pretreatment strategy, strain selection, and process design. The main weakness is that this flexibility often entails additional processing steps, including hydrolysis, supplementation, detoxification, product separation, and quality control. In economic terms, yeast fermentation must also compete with alternative SCG valorization routes, such as extracting antioxidants, oils, dietary fiber, or functional ingredients [3,10,61].

## 8. Comparative Assessment of Microbial Systems

Biological routes for coffee-waste valorization differ substantially in their substrate requirements, product profiles, operational complexity, cost structures, and technological maturity. In this review, the systems were compared based on the type of coffee-derived substrate, the main process objective, reported or prospective products, efficiency indicators, major limitations, cost-related bottlenecks, and coffee-specific maturity level.

Microalgae are most relevant for liquid coffee-derived streams, particularly coffee wastewater and liquid digestates [49,58]. Their main advantage is the ability to combine nutrient removal with biomass generation [58]. However, their performance is strongly dependent on medium conditioning, light penetration, dilution, pH adjustment, contamination control, and biomass harvesting. Therefore, in coffee-specific contexts, microalgal systems are still best described as laboratory-to-early-pilot approaches rather than established industrial solutions [38,43,60].

Bacterial-based systems are predominantly investigated using Pseudomonas and are more specialized and treatment-oriented. Their main strength lies in the degradation of caffeine and the partial reduction in the pollutant load in coffee wastewater. These systems are attractive when the dominant objective is detoxification rather than product diversification. However, their commercial value is limited because the main output is treated or partially detoxified effluent rather than a high-value product. Their broader implementation will require validation with real, compositionally variable coffee-processing wastewaters and careful control of aeration, agitation, biomass loading, and process stability [42,51].

*Pleurotus* cultivation on SCG-containing substrates currently represents the most practically approachable route among the microbial systems considered here [45]. It produces a tangible food product, can be integrated into local circular-economy models, and is already represented by small-scale commercial initiatives. Nevertheless, SCG usually performs better as a component of the substrate [65] than as the sole substrate, and excessive SCG proportions may inhibit mycelial development or fruiting [48]. This route, therefore, still requires substrate formulation, moisture control, pasteurization or sterilization, contamination management, and stable handling of the feedstock [31,41,77].

**Figure 4:**
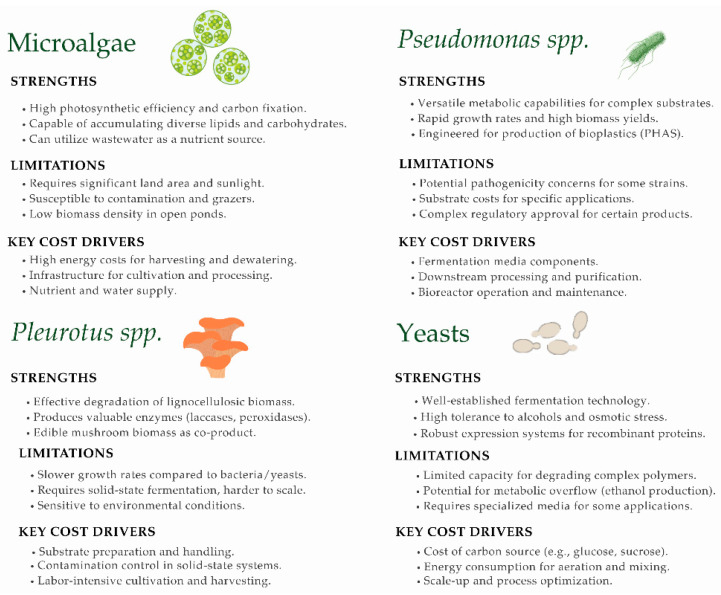
Strengths, limitations, and key cost drivers of selected microorganisms used in spent coffee grounds valorization. Figure 4 summarizes the strengths, limitations, and key cost drivers of microalgae, *Pseudomonas* spp., *Pleurotus* spp., and yeasts across different coffee-derived substrates and residues. Created by the authors using Canva.

Yeast-based systems offer the broadest product portfolio, including ethanol, fermented beverages, aroma-related products, and selected specialty metabolites [68,74]. Their flexibility is a major advantage, particularly when hydrolyzed SCG, coffee husk hydrolysates, mucilage-derived media, or sugar-rich residues are available [46,70]. However, yeast processes are often constrained by the need for residue hydrolysis, nutrient supplementation, inhibitor control, and downstream product recovery [46]. Economically, they may also compete with alternative SCG upcycling routes, such as extracting antioxidants, oils, dietary fiber, or functional ingredients [72]. To complement the detailed comparison presented in Table 4, Figure 4 provides a concise visual summary of the major strengths, limitations, and key cost-related factors associated with the selected microbial groups discussed in this review. Figure 4 is not intended to indicate that all organisms are used under identical coffee-waste conditions, but rather to synthesize their relevance across different coffee-derived substrates and residues, including coffee wastewater, liquid digestates, hydrolyzed SCG, SCG oil, and SCG-containing solid substrates.

Overall, no single microbial system can be considered universally superior. Liquid coffee-derived streams are best suited for treatment-oriented systems such as microalgal cultivation and bacterial bioremediation. In contrast, solid and hydrolyzed residues are better suited for fungal cultivation and yeast fermentation. The most appropriate route depends on the main objective: wastewater treatment, food production, biofuel generation, specialty metabolite production, or integrated biorefinery development.

Table 4 shows that the most suitable microbial route for coffee-waste valorization depends primarily on the physical form and composition of the coffee-derived stream. Liquid streams are better suited to treatment-oriented systems, whereas solid and hydrolyzed residues are more appropriate for product-oriented fungal and yeast processes. From a maturity perspective, *Pleurotus* cultivation on SCG-containing substrates is the most practically advanced coffee-specific route. At the same time, microalgal and *Pseudomonas*-based systems remain more dependent on pilot-scale validation. Yeast-based processes occupy an intermediate position: they offer broad product flexibility, but their economic feasibility depends strongly on efficient hydrolysis, inhibitor management, and downstream recovery.

## 9. Patents, Commercialization, and Regulatory Considerations

As described in Section 2, patent documents were analyzed separately from the peer-reviewed literature. They were used only to identify technological trends and examples of applied innovation, rather than as evidence of experimentally validated process performance. The patent landscape shows that innovation around coffee residues extends beyond fermentation alone and increasingly includes functional ingredients, environmental materials, solid-state fermentation, and integrated valorization concepts.

Singh et al. reported the transformation of spent coffee grounds into microrobots for water treatment [78]. A related U.S. patent application, US20250017234A1, “Coffee ground-derived microbots”, was subsequently published [79]. This related patent application further demonstrates that spent coffee grounds (SCG) are being explored as functional environmental materials for removing contaminants from aqueous media [79]. Another U.S. patent application, US20250255922A1, describes a process for converting SCG into antioxidant dietary fiber via combined microwave-assisted and enzymatic treatment, highlighting the growing interest in the food-ingredient-oriented valorization of coffee residues [80]. These examples suggest that coffee residues are increasingly viewed not only as low-cost substrates for microbial conversion but also as chemically rich feedstocks for advanced materials and functional products. Patent documents related to microbial and fungal processing of coffee are also available. U.S. patent application US20100239711A1 [81] describes solid-state fermentation of coffee with fungi to modify beverage properties, while the Korean patent KR101994276B1 [82] concerns solid-state fermented coffee prepared using microorganisms and reports compositional and sensory modification as key objectives [82]. These examples are directly relevant to microbial biotechnology, although they are more closely associated with product modification and sensory differentiation than with waste treatment alone.

Overall, the patent record supports two broad trends. First, SCG and other coffee residues are increasingly treated as sources of functional ingredients, sorbents, environmental materials, and industrial feedstocks. Second, microbial and solid-state fermentation technologies are being developed not only for waste management but also for sensory modification, product differentiation, and higher-value applications. However, only part of this patent activity directly overlaps with the microbial cultures considered in this review, namely microalgae, bacteria, *Pleurotus*, and yeasts. This indicates that coffee-residue innovation is broader than microbial biotechnology alone.

Commercial implementation of coffee-residue valorization remains uneven across microbial systems. The most tangible and practically advanced route is currently the local circular-economy model, based on the collection of spent coffee grounds and their use in mushroom cultivation. This model benefits from relatively simple logistics, visible food products, and compatibility with urban sustainability initiatives. PermaFungi (Brussels, Belgium) is a well-known example of this approach, using SCG to support oyster mushroom production and circular-economy education [77].

At medium and larger scales, commercialization is shifting toward integrated SCG biorefineries rather than single-product valorization. EcoBean (Warsaw, Poland) represents this direction by positioning spent coffee grounds as an industrial feedstock for multiple product fractions rather than merely as a waste stream, composting material, or low-grade fuel precursor [83,84]. Such models are better suited to high-volume, relatively centralized SCG streams, including residues from industrial coffee processing, instant coffee production, large catering systems, and organized urban collection networks.

However, commercialization remains strongly dependent on feedstock logistics and substrate quality control. Dispersed SCG produced by households, cafés, restaurants, and small retailers may differ substantially in moisture content, storage time, contamination levels, particle size, brewing method, and the presence of non-coffee contaminants. These factors can increase the costs of collection, drying, stabilization, transport, and preprocessing. Therefore, the apparent low cost of coffee residues should not be taken as automatic evidence of economic feasibility.

Among the systems reviewed in this article, *Pleurotus* cultivation on SCG-containing substrates appears closest to practical implementation, especially in local and small-scale circular models.

Regulatory requirements for coffee-derived products depend strongly on the intended final application. Outputs intended for food, feed, fertilizers, cosmetics, technical materials, wastewater treatment, or bioenergy cannot be treated under a single regulatory pathway. This distinction is particularly important because the same coffee-derived residue may be acceptable for one application but unsuitable, or legally more demanding, for another.

Within the European Union, coffee-derived ingredients intended for food applications may fall under Regulation (EU) 2015/2283 on novel foods if they were not consumed to a significant degree in the EU before 15 May 1997 [85]. Feed applications are governed by Regulation (EC) No 767/2009, while Commission Regulation (EU) No 68/2013 establishes the Catalog of feed materials [86,87]. Consequently, future market uptake will depend not only on technical feasibility but also on product classification, safety documentation, labeling, traceability, contaminant control, and compliance with food or feed regulations.

Safety assessment is particularly important for products derived from SCG, coffee pulp, husk, silverskin, or fermented coffee residues intended for food or feed use. Key concerns include microbial contamination, fungal growth, mycotoxins, pesticide residues, heavy metals, residual caffeine, phenolic compounds, storage stability, and batch-to-batch variability. These risks are especially relevant for wet SCGs, which are highly perishable and may deteriorate rapidly if not dried, stabilized, or processed under hygienic conditions.

For microbial bioprocesses, additional safety issues may arise from the selected organism, cultivation conditions, and final product category. Edible fungi such as *Pleurotus* spp. may be suitable for food-oriented applications when grown under controlled, hygienic conditions. In contrast, bacterial or algal systems used for wastewater treatment are more likely to produce outputs that require further treatment, are restricted in use, or are intended for technical rather than food-related applications. Similarly, yeast-based processes may be directed toward food, beverage, bioethanol, or specialty metabolite production, but each route requires different quality and safety specifications.

Despite promising results, *Pseudomonas*-based bioremediation of coffee wastewater remains constrained by several limitations. First, much of the strongest evidence comes from synthetic or controlled wastewater systems, where caffeine concentration, pH, and matrix composition are easier to manage than in real industrial effluents. Second, caffeine degradation does not necessarily imply complete wastewater remediation because COD, BOD, nutrients, solids, phenolics, and toxicity may require additional treatment steps.

Third, the process value is mainly treatment-oriented rather than product-oriented. Unlike *Pleurotus* cultivation, which produces edible biomass, or yeast fermentation, which can generate ethanol or specialty metabolites, *Pseudomonas*-based systems primarily generate treated or partially detoxified effluent. This does not reduce their environmental relevance, but it affects the logic of commercialization and cost recovery.

Fourth, scale-up requires stable operation under variable wastewater composition, sufficient aeration, control of biomass concentration, and management of mixed microbial populations. These requirements may increase operational complexity and cost, particularly if the process is implemented outside controlled laboratory conditions.

*Pseudomonas* spp. are among the most credible bacterial candidates for the bioremediation of caffeine-rich coffee wastewater because they can degrade caffeine, a characteristic inhibitory compound in coffee-derived liquid streams. However, their coffee-specific technological maturity remains limited. The strongest evidence supports their use in synthetic or controlled wastewater systems and early bioreactor studies, while real-effluent applications and integrated treatment trains still require further validation.

Therefore, regulatory-oriented product development should begin at the process-design stage. Coffee-derived microbial products should be developed with a clearly defined target category, such as food ingredient, feed component, fertilizer, cosmetic ingredient, technical sorbent, biofuel precursor, or wastewater-treatment output. This would reduce the risk of producing technically promising materials that cannot realistically enter the market due to safety, labeling, or regulatory barriers.

The main barriers to broader implementation include substrate heterogeneity, unstable supply chains, high moisture content of fresh SCG, storage-related contamination, pretreatment costs, lack of standardized residue characterization, uncertain product classification, and limited pilot-scale validation. Future commercialization will therefore require not only improved microbial performance, but also reliable logistics, quality-control protocols, techno-economic assessment, life-cycle assessment, and early alignment with regulatory requirements.

In this context, coffee residues should be regarded as promising but application-specific feedstocks. Their successful use in microbial biotechnology will depend on matching the right residue stream with the right microorganism, process design, product category, and regulatory pathway. No single system is universally superior; rather, each route occupies a different niche within coffee-waste valorization.

## 10. Practical Perspectives and Future Research Directions

One of the most credible future directions is the design of cascading coffee biorefineries in which different microorganisms treat distinct fractions of the coffee residue stream [15]. In such a configuration, microalgae could polish coffee-derived liquid streams after suitable conditioning [38,49]. Anaerobic digestion could recover energy from liquid or residual biomass fractions [60], and *Pleurotus* cultivation could valorize selected solid residues such as SCG-containing blends [48]. Yeast fermentation could also be integrated after the hydrolysis of carbohydrate-rich fractions [46], while residual biomass could be further directed to composting [21], anaerobic digestion, or material applications. This concept is well aligned with current biorefinery logic, although fully integrated and experimentally validated coffee-residue process chains remain rare [43].

**Figure 5:**
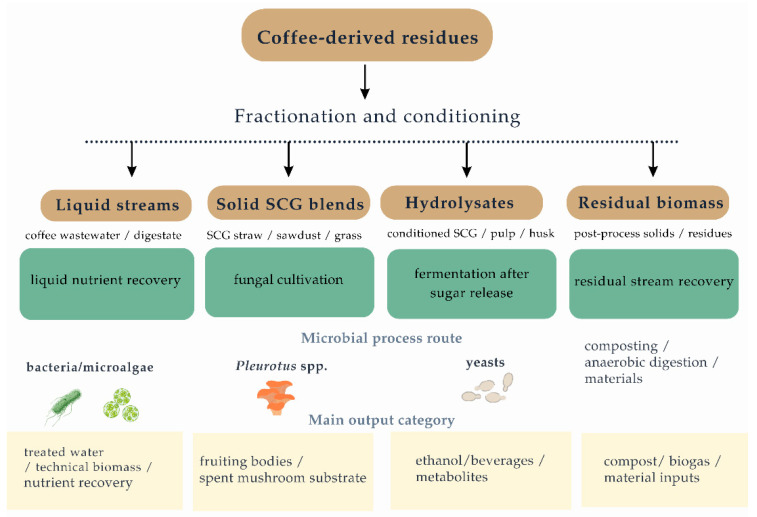
Proposed cascading biorefinery model for coffee-residue valorization. Figure 5 summarizes a stream-specific approach in which coffee-derived residues are first fractionated or conditioned and then assigned to microbial or process routes based on their physical form, composition, inhibitor profile, and realistic output value. Liquid streams are linked mainly to bacterial or microalgal routes; solid SCG-based blends to fungal cultivation; hydrolysates to yeast-based fermentation; and residual biomass to composting, anaerobic digestion, or material recovery. Created by the authors using Canva.

A proposed cascading framework for matching coffee-derived residue fractions with suitable microbial routes and output categories is shown in Figure 5.

Future projects should also explore mixed and sequential microbial systems rather than relying solely on single-organism processes [37,56]. Coffee-derived residues contain complex mixtures of lignocellulosic polymers, soluble sugars, lipids, caffeine, tannins, and phenolic compounds [1,15], which are unlikely to be optimally converted by a single microorganism under a single set of conditions. Sequential processes, such as bacterial detoxification coupled with microalgal polishing [38,63], fungal pretreatment followed by yeast fermentation [31,32,44], or anaerobic digestion followed by algal nutrient recovery [49,60], may improve overall substrate utilization and reduce inhibitory effects. However, such systems require careful control of microbial compatibility, contamination, process timing, and product safety.

A major priority for future research is the standardization of coffee-derived substrates. Many studies report promising results using SCG, coffee wastewater, pulp, husk, silverskin, or digestate, but differences in residue origin, coffee species, roasting degree, brewing method, moisture content, storage time, particle size, and contamination levels limit direct comparisons [2,12,25]. Future studies should report a minimum characterization set, including moisture, pH, C/N ratio, total solids, volatile solids, ash, carbohydrate, lipid, protein, caffeine, and total phenolic content [4,15]. For liquid streams, COD, BOD, ammonium, phosphate, suspended solids, color, and pH should also be reported [37,38]. Such standardization would improve reproducibility and enable meaningful comparisons across microbial systems.

Future research should move beyond proof-of-concept microbiology toward pilot-scale systems with realistic feedstock logistics and repeated production cycles [43]. Although coffee residues are often described as low-cost substrates, their practical use entails costs for collection, transport, drying, storage, pretreatment, sterilization or pasteurization, hydrolysis, detoxification, and downstream product recovery. These costs may outweigh the apparent advantage of using a waste-derived feedstock, especially in microalgal and yeast-based systems that require substantial conditioning or harvesting.

Techno-economic assessment should therefore become a routine component of future studies. Coffee-based media and substrates should be compared not only with untreated waste disposal scenarios, but also with conventional microbial substrates and alternative valorization routes [9]. Life-cycle assessment is equally important because the environmental benefits of coffee-waste valorization may be reduced by energy-intensive pretreatment, drying, artificial lighting, aeration, or harvesting [6]. Future LCA studies should compare microbial valorization routes with composting, anaerobic digestion, thermochemical conversion, material recovery, and landfilling.

At a small scale, the most realistic business model remains local circularity centered on SCG collection and mushroom cultivation. Permafungi is a well-known example of this approach, using spent coffee grounds to support oyster mushroom production and circular-economy education. At medium and larger scales, the more promising model is the integrated SCG biorefinery. EcoBean is a relevant example, positioning itself as a technology provider for converting spent coffee grounds into multiple product fractions and presenting SCG not merely as a fuel precursor but as a feedstock for industrial circular manufacturing [77,83,84].

However, commercial implementation will depend on stable feedstock supply, substrate quality control, predictable product specifications, and realistic logistics. The feasibility of coffee-residue valorization may differ substantially between centralized industrial streams, such as instant coffee production residues, and dispersed urban SCG generated by households, cafés, and restaurants. This distinction should be considered in future process design and business-model assessment.

## 11. Conclusions

Coffee processing residues and spent coffee grounds should not be regarded as universal ready-to-use microbial media, but rather as adaptable bioresources that can serve as substrates, co-substrates, or medium components for selected microorganisms after appropriate conditioning. The reviewed evidence indicates that coffee-derived residues are most convincingly applicable in four main directions: fungal cultivation on SCG-containing substrates [41,44,48,65], bacterial biodegradation of caffeine-rich wastewaters [42,51,62], yeast-based fermentation of hydrolyzed residues [46,68,70], and microalgal treatment of conditioned liquid streams [38,58,60]. These routes demonstrate that coffee residues can be repurposed from disposal burdens into feedstocks for bioremediation, biomass generation, food production, and the development of integrated biorefineries.

The comparative assessment also shows that no single microbial system is universally superior. Despite this potential, coffee-based microbial bioprocessing remains limited by substrate heterogeneity, inhibitory compounds, pretreatment requirements, scale-up challenges, and downstream processing costs [1,15,75]. Commercial maturity is therefore uneven across systems, with fungal cultivation more advanced in practice, while microalgal, bacterial, and many yeast-based applications remain largely at the laboratory or pilot scale. Future development should focus on residue standardization, integrated multi-step valorization, techno-economic and life-cycle assessment [9,15], and compliance with food, feed, safety, and regulatory requirements [17]. These steps will be essential if coffee-derived residues are to move from promising experimental substrates to reliable components of sustainable microbial biotechnology.

## Figures and Tables

**Table 1:** General classification of primary and secondary residues generated during coffee processing and consumption.

Category	Residue	Origin/Processing Stage	Key Characteristics	References
Primary residues	Coffee pulp	Wet processing: separation of the bean from the fruit	Largest fraction; rich in lignocellulosic material	[3,12,16,17]
	Coffee husk	Wet processing: separation of the bean from the fruit	Contains outer dried fruit layers; high fiber content	
	Mucilage	Intermediate processing stages	Sticky carbohydrate-rich layer removed during fermentation/demucilation	
	Parchment	Intermediate processing stages	Structurally rigid; high cellulose and lignin content	
	Coffee leaves	Agricultural biomass from coffee plants	Source of caffeine and mangiferin	
	Coffee flowers	Agricultural biomass from coffee plants	Contains bioactive compounds	
	Coffee processing wastewater	Wet processing and demucilization	Liquid stream rich in organic matter	
Secondary residues	Spent Coffee Grounds	Brewing and coffee consumption	Most abundant secondary residue; contains lipids, fiber, nitrogen compounds, and bioactives	[3,6,11,13,18]
	Silverskin	Roasting process	Thin papery layer; rich in fiber andantioxidants	

**Table 2:** Suitability of coffee-derived residues for selected microbial systems.

Coffee-Derived Residue	Main Useful Components	Main Limitations	Most Suitable Microbial Systems	Typical Role	References
SCG	Lipids, hemicellulose, proteins, residual sugars	Caffeine, phenolics, moisture, lignocellulosic recalcitrance, variability	*Pleurotus*, yeasts after hydrolysis, selected bacteria, and oleaginous microorganisms	Solid substrate, hydrolysate, lipid-rich feedstock	[30,50]
Coffee wastewater	Soluble organics, nitrogen, phosphorus, dissolved nutrients	Acidity, high COD/BOD, caffeine, phenolics, dark color	*Pseudomonas*, adapted bacteria, and microalgae after conditioning	Liquid medium, remediation stream	[42,51]
Coffee pulp	Sugars, cellulose, polyphenols, and moisture	Rapid spoilage, high moisture, and phenolic inhibition	Yeasts, fungi, bacteria	Fermentable residue, fungal substrate, composting feedstock	[52,53]
Coffee husk/parchment	Cellulose, lignocellulose, minerals	Lignin, tannins, structural recalcitrance	Fungi, yeasts, or bacteria after hydrolysis	Solid substrate, hydrolysate, lignocellulosic feedstock	[17,54,55,56]
Silverskin	Fiber, antioxidants, residual proteins	Variable composition, lower mass availability, fibrous matrix	Fungi, bacteria, food/feed-related microbial applications	Additive, co-substrate, source of bioactives	[57]
Digestate	Ammonium nitrogen, phosphorus, minerals, dissolved nutrients	Dark color, turbidity, ammonium inhibition, residual COD	Microalgae after pretreatment, algal–bacterial systems	Nutrient-rich liquid stream, wastewater-polishing medium	[38,39,49,58]

**Table 3:** Selected yeasts used or proposed for coffee-residue fermentation.

Yeast Species	Coffee-Derived Substrate	Required Conditioning	Main Products	Maturity/Limitation
*S. cerevisiae*	hydrolyzed SCG, husk hydrolysate	hydrolysis, nutrient balancing	ethanol, metabolites	most established
*P. kluyveri*	SCG hydrolysate	hydrolysis, supplementation	low-alcohol beverages, aroma compounds	exploratory
*K. marxianus*	prospective hydrolysates	sugar release, inhibitor control	biomass, ethanol, enzymes	promising but limited coffee-specific data
*Y. lipolytica*	lipid-rich SCG fractions	extraction/conditioning	lipids, organic acids, aroma-related products	conceptually relevant, limited direct validation

**Table 4:** Comparative assessment of microbial systems used for coffee-waste valorization: substrates, products, efficiency indicators, bottlenecks, maturity, and representative sources.

Microbial System	Main Coffee-Derived Substrate	Primary Value Logic	Main Products or Outputs	Main Efficiency Indicators	Coffee -Specific Maturity	References
Microalgae	Coffee wastewater, liquid digestate, and, less commonly, SCG-derived fractions	Treatment-oriented and biomass-oriented	Treated water, technical algal biomass, lipids for biofuels, anaerobic digestion feedstock, fertilizer precursors; food/feed/pigment applications require separate safety validation.	Nutrient removal, biomass productivity, COD reduction, and color reduction after pretreatment	Low to medium; mostly laboratory and early pilot scale in coffee-specific applications	[38,43,60]
*Pseudomonas* spp.	Coffee wastewater, especially caffeine-containing liquid streams	Treatment-oriented	Detoxified or partially remediated wastewater; caffeine removal	Caffeine degradation, COD reduction, BOD reduction, TOC reduction	Low to medium; mainly laboratory and controlled bioreactor studies	[37,42,51]
*Pleurotus* spp.	SCG-containing mixed substrates, usually with sawdust, straw, or other lignocellulosic co-substrates; SCG is more reliable as a supplement than as the sole substrate.	Food/ product-oriented	Edible mushrooms, spent mushroom substrate, and compost-like residual material.	Biological efficiency, yield, mycelial growth rate, fruiting success, caffeine reduction	Medium to high compared with other coffee-specific microbial routes; a practical small-scale implementation exists.	[15,41,48,77]
Yeasts	Hydrolyzed SCG, coffee husk hydrolysates, mucilage-derived media, sugar-rich coffee residues	Fermentation /product-oriented	Ethanol, fermented beverages, aroma compounds, glutathione, 5-aminolevulinic acid, specialty metabolites	Sugar conversion, ethanol yield, product titer, fermentation efficiency, metabolite concentration	Variable; ethanol and beverage-oriented routes are more developed than specialty coffee-specific biorefineries.	[68,71,74]

Note: Maturity levels are presented comparatively and refer to coffee-specific applications rather than to the broader maturity of microalgal cultivation, bacterial wastewater treatment, mushroom cultivation, or yeast fermentation technologies. Downstream use of biomass derived from waste media requires application-specific safety and regulatory assessments. In *Pleurotus* cultivation, SCG is best interpreted as a substrate component rather than a universally suitable sole substrate.

## Data Availability

No new data were created or analyzed in this study. Data sharing does not apply to this article.

## Abbreviations

The following abbreviations are used in this manuscript:

BOD	Biochemical oxygen demand
C/N	Carbon-to-nitrogen ratio
COD	Chemical oxygen demand
EU	European Union
GAE	Gallic acid equivalents
LCA	Life-cycle assessment
NCIM	National Collection of Industrial Microorganisms
PHA	Polyhydroxyalkanoates
PRISMA	Preferred Reporting Items for Systematic Reviews and Meta-Analyses
SCG	Spent coffee grounds
spp.	Species pluralis
sp.	Species singularis
SSF	Solid-state fermentation
TOC	Total organic carbon
